# Effective therapy of the small-molecule cocktail 5SM on adult rat heart after ischemia–reperfusion injury

**DOI:** 10.1093/jmcb/mjad034

**Published:** 2023-05-31

**Authors:** Lixia Zheng, Yuanyuan Chen, Zhengyuan Wu, Xiaojun Zhu, Jing-Wei Xiong

**Affiliations:** Beijing Key Laboratory of Cardiometabolic Molecular Medicine, Institute of Molecular Medicine, College of Future Technology, and State Key Laboratory of Natural and Biomimetic Drugs, Peking University, Beijing 100871, China; PKU-Nanjing Institute of Translational Medicine, Nanjing 211800, China; Beijing Key Laboratory of Cardiometabolic Molecular Medicine, Institute of Molecular Medicine, College of Future Technology, and State Key Laboratory of Natural and Biomimetic Drugs, Peking University, Beijing 100871, China; PKU-Nanjing Institute of Translational Medicine, Nanjing 211800, China; PKU-Nanjing Institute of Translational Medicine, Nanjing 211800, China; Beijing Key Laboratory of Cardiometabolic Molecular Medicine, Institute of Molecular Medicine, College of Future Technology, and State Key Laboratory of Natural and Biomimetic Drugs, Peking University, Beijing 100871, China; PKU-Nanjing Institute of Translational Medicine, Nanjing 211800, China; Beijing Key Laboratory of Cardiometabolic Molecular Medicine, Institute of Molecular Medicine, College of Future Technology, and State Key Laboratory of Natural and Biomimetic Drugs, Peking University, Beijing 100871, China; PKU-Nanjing Institute of Translational Medicine, Nanjing 211800, China

**Dear Editor**,

Myocardial infarction (MI) is one of the leading causes of cardiovascular-related mortality worldwide. Timely restoration of the blood supply to ischemic myocardium by thrombolysis or percutaneous coronary intervention is a common clinical practice and decreases the mortality risk for MI patients (Heusch, 2020). However, restoring myocardial perfusion within a certain period of time causes further injury, known as myocardial ischemia–reperfusion (IR) injury, that induces cardiac dysfunction and eliminates the benefits of reperfusion therapy (Heusch, 2020; Hausenloy and Yellon, 2013). Because currently no clinical practices can reverse the massive loss of cardiomyocytes (CMs) after MI, the field holds great hope for cardiac regenerative medicine (Sadek and Olson, 2020). We recently discovered a chemical cocktail of five small molecules (5SM) that promotes heart regeneration by inducing CM proliferation through activating lactate–LacRS2–mTOR signaling after permanent MI in rats (Du et al., 2022). To address their clinical relevance, we examined whether 5SM confers similar benefits on the rat heart after IR injury.

To address the therapeutic role of 5SM in rat IR, we administered dimethylsulfoxide (DMSO) or 5SM immediately after IR (acute IR) or at 7 days post-IR (subacute IR). Adult rats were subjected to a 60-min occlusion of the left anterior descending coronary artery followed by reperfusion as previously described (Zhang et al., 2016). We confirmed comparable ischemic and infarcted areas between the DMSO and 5SM groups by Alcian blue and triphenyl tetrazolium chloride staining (Supplementary Figure S1). In the acute IR model, we smeared GelMA hydrogel containing DMSO or 5SM on hearts immediately after ligation and injected DMSO or 5SM intraperitoneally (Figure 1A; Supplementary Figure S2) and then assessed cardiac function by echocardiography (ECHO) at 3, 7, 28, and 56 days post-IR. Importantly, 5SM treatment improved cardiac function and structure [ejection fraction (EF), fractional shortening (FS), left ventricular anterior systolic wall thickness (LVAW;s), and left ventricular posterior systolic wall thickness (LVPW;s)] at 7, 28, and 56 days post-IR (Figure 1B–D; Supplementary Figure S3A and B). Masson's staining showed that 5SM ameliorated cardiac fibrosis compared with DMSO at 8 weeks post-IR (Figure 1E). In addition, at 7 days post-IR, 5SM treatment increased the numbers of Ki67^+^ and pH3^+^ CMs in the infarct border zone of left ventricles, decreased Ki67^+^/vimentin^+^ fibroblasts, and increased CD31^+^ blood vessels (Figure 1F–J). 5SM did not have an anti-apoptotic effect on CMs, by performing LDH detection at 24 h post-IR and TUNEL staining at 7 days post-IR (Figure 1K and L). In the subacute IR model, we delivered DMSO or 5SM at 7 days post-IR (Figure 1M). ECHO showed that 5SM evidently improved cardiac function and structure (EF, FS, LVAW;s, and LVPW;s) at 14 days post-IR and improved cardiac function during the 8 weeks of therapy compared with DMSO (Figure 1N–P; Supplementary Figure S3C and D). Direct comparison between acute IR and subacute IR showed similar changes in cardiac function at different time points (Supplementary Figure S4). Consistently, Masson's staining showed that 5SM decreased infarction size and fibrosis compared with DMSO at 9 weeks post-IR (Figure 1Q). Our previous work showed that 5SM treatment results in notable improvements in heart function and structure in the acute MI group (Du et al., 2022). Similarly, 5SM treatment improved cardiac function in the subacute MI group (Supplementary Figure S5A–D) and ameliorated infarction size and fibrosis (Supplementary Figure S5E and F). Notably, 5SM treatment did not evidently affect the morphology of the heart, liver, lung, and kidney or the organ weight ratio of rats compared with DMSO (Supplementary Figure S6).

**Figure 1:**
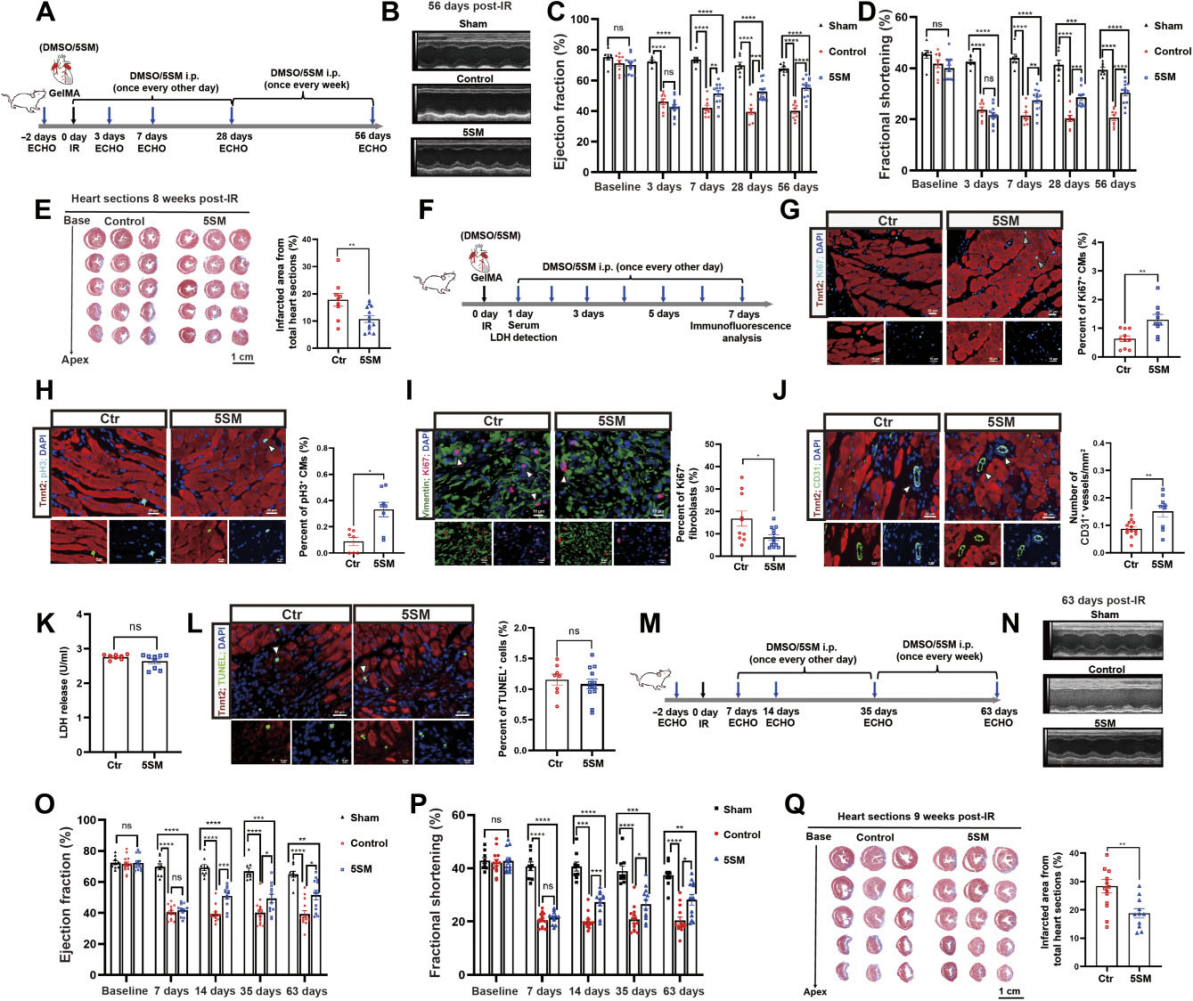
5SM improves cardiac function and decreases cardiac fibrosis via increasing CM proliferation and angiogenesis whereas decreasing fibroblast proliferation after IR injury. (**A**–**E**) 5SM improved cardiac function and decreased cardiac fibrosis after acute IR injury. (**A**) Schematic showing DMSO or 5SM delivery, ECHO, and heart harvests in adult rats after acute IR. (**B**) Representative images of M-mode ECHO from sham, control (DMSO-treated), and 5SM-treated rats at 56 days post-IR. Heart rates in three groups were controlled to be similar. (**C** and **D**) Serial ECHO measurements of ejection fraction and fractional shortening in rats after acute IR. (**E**) Masson's trichrome staining of heart sections from control and 5SM-treated rats at 56 days post-IR and quantitative analysis of cardiac fibrosis. The fibrotic scar area was calculated relative to the total section area. Sham, *n* = 7; control, *n* = 9; 5SM, *n* = 13. Data are presented as mean ± SEM; ns, not significant; **P < 0.01; ***P < 0.001; ****P < 0.0001; two-way ANOVA followed by Tukey's multiple comparisons test (**C** and **D**) or unpaired, two-tailed Student's *t*-test (**E**). Scale bar, 1 cm. (**F**–**L**) 5SM increased CM proliferation and angiogenesis, decreased fibroblast proliferation, but did not affect cell apoptosis after acute IR injury. (**F**) Schematic showing DMSO or 5SM delivery, serum LDH detection, and immunofluorescence analysis in adult rats within 7 days after acute IR. (**G**–**J** and **L**) Representative images and quantification of Ki67^+^ CMs (**G**; control, *n* = 5; 5SM, *n* = 4), pH3^+^ CMs (**H**; control, *n* = 4; 5SM, *n* = 5), CD31^+^ blood vessels (**J**; *n* = 5 per group), and TUNEL^+^ cells (**L**; *n* = 5 per group) in the border zone of infarct left ventricles, as well as Ki67^+^/vimentin^+^ fibroblasts (**I**; *n* = 5 per group) in the infarct area of left ventricles, at 7 days post-IR. (**K**) Serum LDH level at 24 h post-IR. *n* = 5 per group. Data are presented as mean ± SEM; ns, not significant; *P < 0.05; **P < 0.01; unpaired, two-tailed Student's *t*-test. Scale bar, 20 μm (top) and 10 μm (bottom). (**M**–**Q**) 5SM improved cardiac function and decreased cardiac fibrosis after subacute IR injury. (**M**) Schematic showing DMSO or 5SM delivery, ECHO, and heart harvests in adult rats after subacute IR. (**N**) Representative images of M-mode ECHO from sham, control, and 5SM-treated rats at 63 days post-IR. Heart rates in three groups were controlled to be similar. (**O** and **P**) ECHO data showing that 5SM treatment increased ejection fraction and fractional shortening in rats after subacute IR. (**Q**) Masson's trichrome staining of serial heart sections from control and 5SM-treated rats at 63 days post-IR and quantitative analysis of cardiac fibrosis. Sham, *n* = 9; control, *n* = 14; 5SM, *n* = 12. Data are presented as mean ± SEM; ns, not significant; *P < 0.05; **P < 0.01; ***P < 0.001; ****P < 0.0001; two-way ANOVA followed by Tukey's multiple comparisons test (**O** and **P**) or unpaired, two-tailed Student's *t*-test (**Q**). Scale bar, 1 cm.

It becomes well recognized that induction of CM reentry into the cell cycle and cytokinesis can promote heart regeneration. Our previous work demonstrated that 5SM promotes adult rat heart regeneration via induction of CM proliferation after acute MI (Du et al., 2022). In this work, we found that 5SM shows beneficial effects by improving cardiac function and decreasing cardiac fibrosis in acute IR and subacute MI/IR models. This warrants future studies on both elucidating the fundamental mechanisms by which 5SM promotes heart regeneration and developing chemical-induced heart regeneration strategies for treating ischemic heart diseases.

*We would like to acknowledge Prof. Iain C. Bruce, Guest Professor at Peking University, for his critical comments and revisions of this manuscript. This work is supported by grants from the National Key R&D Program of China (2018YFA0800501 and 2019YFA0801602), the National Natural Science Foundation of China (32230032, 31730061, 31430059, and 81870198), and Synogen Biopharma Co., Nanjing, China. L.Z. and Y.C. designed and performed experiments, analyzed data, and wrote the manuscript; Z.W. helped with ECHO; X.Z. supervised this work and revised this manuscript; J.-W.X. conceived, supervised, and designed this work, analyzed the data, and revised this manuscript. The featured image was created with biorender.com.]*

## Supplementary Material

mjad034_Supplemental_File
