# Supplementary material for: Effective therapy of the small-molecule cocktail 5SM on adult rat heart after ischemia–reperfusion injury
Source: J Mol Cell Biol. 2023 May 31;15(6):mjad034. doi: 10.1093/jmcb/mjad034 (PMC10701712; doi:10.1093/jmcb/mjad034)
Supplement: mjad034_Supplemental_File [file mjad034_supplemental_file.pdf]

Supplementary material

Effective therapy of the small-molecule cocktail 5SM on adult rat heart after ischemia-reperfusion injury

Lixia Zheng[1,2,†], Yuanyuan Chen[1,2,†], Zhengyuan Wu[2], Xiaojun Zhu[1,2], and Jing-Wei Xiong[1,2,*]

[1]Beijing Key Laboratory of Cardiometabolic Molecular Medicine, Institute of Molecular Medicine, College of Future Technology, and State Key Laboratory of Natural and Biomimetic Drugs, Peking University, Beijing 100871, China;

[2]PKU-Nanjing Institute of Translational Medicine, Nanjing 211800, China;

† These two authors contributed equally to this work

*Corresponding to: Jing-Wei Xiong, Ph.D., Professor,

Email: jingwei_xiong@pku.edu.cn

Supplementary Methods

Animals

Adult (8 week-old) male wild-type Sprague-Dawley (SD) rats were purchased from Vital River Laboratory Animal Technology Co., Ltd (Beijing, China). All procedures involving experimental rats were performed according to the animal protocols approved by the Institutional Animal Care and Use Committee at Peking University, Beijing, China.

IR and MI model

Adult rat was anesthetized by tribromoethanol (300 mg/kg; Sigma) and connected to a rodent ventilator (MouseVent, Kent Scientific Corp., Torrington, CT, USA). A thoracotomy was made and the heart was exposed by removal of the pericardium. In IR model, the LAD coronary artery was ligated using a 6-0 nylon suture for 1h and then losing it. In MI model, the LAD coronary artery was permanently ligated. The thoracic cavity and skin were immediately closed with 4-0 suture. Sham group was subjected to the same procedures except that the suture was passed under the LAD but not tied.

Delivery of DMSO and 5SM into rats after IR/MI

In acute IR group, 5SM (PE, 20 μmol/L; BAR, 40 μmol/L; HAR, 50 μmol/L; VOO, 20 μmol/L; AZD, 20 μmol/L) or the same dose of DMSO were dissolved in GelMA hydrogel and smeared on the border zone of infarct area immediately after ligation, and used ultraviolet light to coagulate the hydrogel. Then the rats were intraperitoneally injected with 5SM (PE: 2 mg/kg, BAR: 2 mg/kg, HAR: 2 mg/kg, VOO: 10 μg/kg, AZD: 2 mg/kg) or the same dose of DMSO once every other day in the first 4 weeks and once a week in the second 4 weeks post-IR. In subacute IR and subacute MI groups, rats were delivered either DMSO or 5SM intraperitoneally the same way as acute IR group except that this treatment was conducted till 7 days after IR.

Echocardiography (ECHO)

ECHO was performed on rats anesthetized with 1% isoflurane, using a high-resolution Vevo 3100 Ultrasound (Visual Sonics) equipped with a 20-MHz variable frequency transducer. Rat hearts were viewed on two-dimensional short-axis planes and data were analyzed on the basis of a

standard formula. All of these data were acquired and analyzed by an investigator who was blinded to the genotypes and drug treatments of the animal groups.

Heart collection and histological analysis

Rats were anaesthetized with tribromoethanol (300 mg/kg; Sigma) and then killed by injection of 10% KCl to the heart apex. Hearts were excised, washed in PBS, and fixed in 4% formaldehyde at room temperature for 48 h. The heart tissue was sectioned at 5 μm and the sections were stained with Masson trichromic solution, and analyzed for the extent of fibrosis under microscopy. The fibrosis area and total section area in each section were measured using ImageJ, and the fibrosis area is reported as a percentage of the total section area.

Alcian blue and triphenyl tetrazolium chloride (TTC) double staining

Fresh hearts were collected and washed with phosphate buffered saline (PBS) three times. Each heart was perfused with 1% Alcian blue (Sigma-Aldrich, A3157) through the aorta. The hearts were then frozen at -20°C at least 30 min and cut into 4 slices according to the ligation site. Slices were then incubated at 37°C in PBS containing 1% TTC (Solarbio, G3005) for 15 min.

Serum lactate dehydrogenase (LDH) detection

Rats were anesthetized by intraperitoneal injection of tribromoethanol (300 mg/kg; Sigma-Aldrich). Blood samples were collected (1 ml/each rat) via the tail vein and placed at room temperature for 1 h prior to centrifugation at 3,000 × rpm for 10 min. Serum was collected and LDH was measured with an assay kit (Beyotime, C0016).

Immunostaining of heart tissues

Fresh hearts were washed with PBS three times and fixed in 4% paraformaldehyde (PFA) at room temperature for 48h. Hearts were then embedded in paraffin and serially sectioned at 5 μm. The sections were dewaxed in xylene, rehydrated in an ethanol series, and washed in PBS. The sections were then rinsed in pre-heated citric acid buffer (pH9.5) and boiled in a microwave for 15 min. After washing with PBS three times, the sections were blocked in PBS containing 1% Tween, 10% FBS and 1% DMSO for 30 min, and then incubated overnight at 4°C with the primary

antibodies: Mouse anti-cardiac troponin T (1:300, Abcam, ab8295), Rabbit phospho-histone H3 (pH3, Ser10) (1:300, CST, 9701S), Rabbit anti-Ki67 antibody (1:500, Abcam, ab15580), mouse anti-Ki67 antibody (1:500, CST, 9449S), Rabbit anti-CD31 antibody (1;300, Abcam, ab134168), Rabbit anti-vimentin antibody (1:300, Abcam, ab92547). For TUNEL staining, heart sections were incubated with TUNEL detection reagent for 1 h (Beyotime, C1088) before primary antibody incubation. After washing with PBS three times, the sections were incubated with the corresponding fluorescence-labeled secondary antibodies: Donkey to Rabbit IgG - H&L (Alexa Fluor® 488) (Abcam, ab150073) or Donkey to Mouse IgG - H&L (Alexa Fluor® 555) (Abcam, ab150110) at room temperature for 2 h. The sections were then counter-stained with DAPI. Images were acquired using Axio Scan Z1 (Zeiss) and analyzed by Zen software (Zeiss).

Hematoxylin/eosin (HE) staining

Fresh hearts were washed with PBS three times and fixed in 4% paraformaldehyde (PFA) at room temperature for 48 h. Hearts were then embedded in paraffin and serially sectioned at 5 μm. The sections were then stained with a Hematoxylin-Eosin (HE) Stain Kit (Solarbio, G1120).

Statistical analysis

All data is presented as mean ± standard error of the mean (SEM). The value of n is mentioned in the figure/figure legends and always stands for separate biological replicates. Statistical analysis was performed using Graphpad Prism 8.3.0. The significant differences between two groups was determined using unpaired Student's t test, with two-tailed P values. Among three or more groups, one-way ANOVA analysis (using the post Dunnett's multiple comparisons test) and two-way ANOVA analysis (using the post Tukey's multiple comparisons test) was used for comparisons. A P value < 0.05 is considered being statistically significant and individual p values are mentioned in the figures and figure legends.

Supplementary Figures

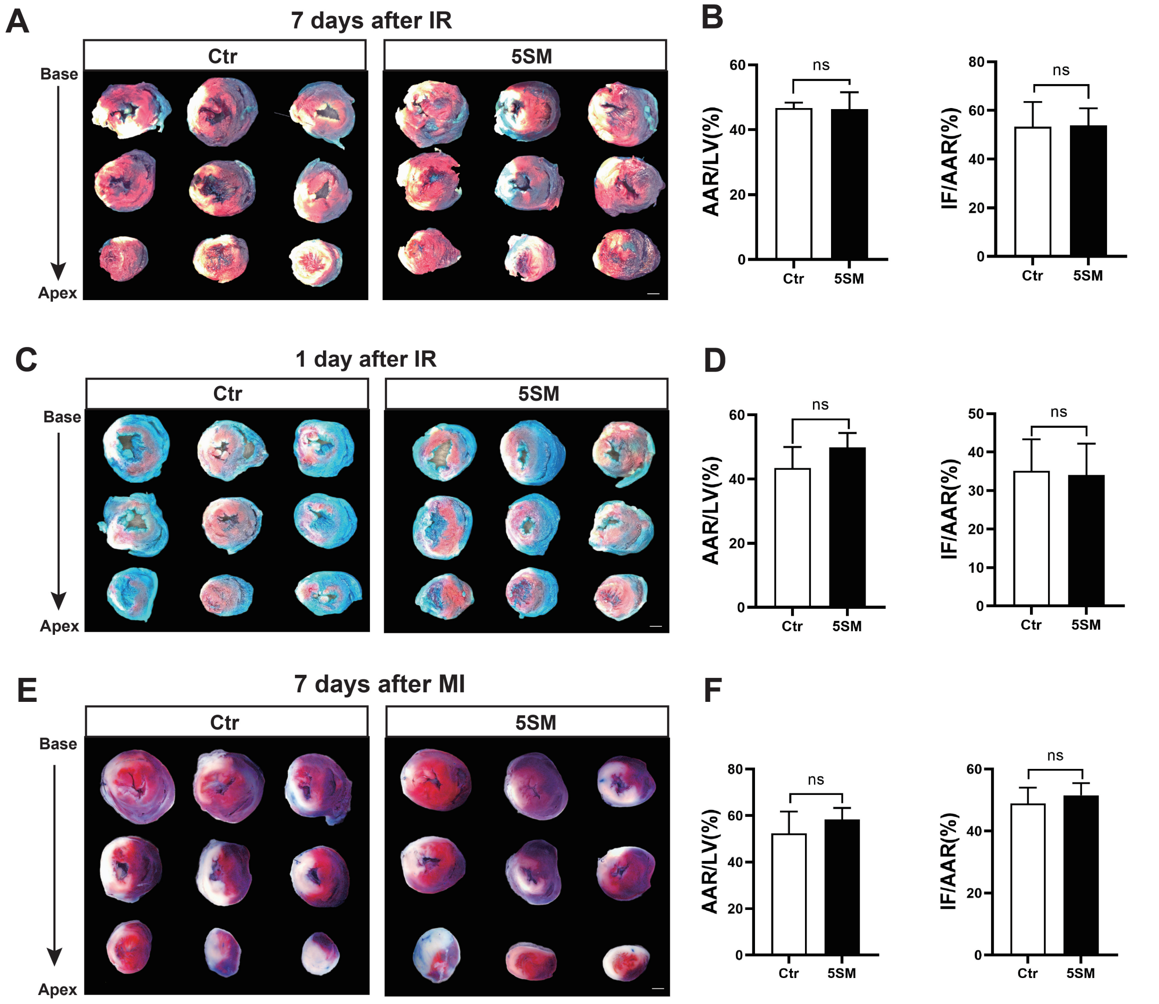

Supplementary Figure S1. Alcian blue and TTC staining showing that the baseline infarct size was comparable among experimental groups. (A) Representative Alcian blue and TTC staining showing comparable ischemic regions between DMSO (Ctr) and 5SM-treated hearts at 7 days after IR. In the ischemic area, the infarct region was stained white and viable cardiac tissue was stained red. Non-ischemic heart muscle was stained blue. Scale bar, 1 mm. **(B)** Quantitative analysis of the data from panel **A**. n = 3 rats/group; Data are mean ± SEM.; ns, no significant; unpaired, two-tailed Student's *t*-test. Ischemic area (area at risk) was calculated as the combined infarct area (white area) and viable area (red area) to the whole left ventricle (LV) size. **(C)** Representative Alcian blue and TTC staining showing comparable ischemic regions between DMSO (Ctr) and 5SM-treated hearts at 1 day after IR. Scale bar, 1 mm. **(D)** Quantitative analysis of the data from panel **C**.

n = 3 rats/group; Data are mean ± SEM.; ns, no significant; unpaired, two-tailed Student's *t*-test. **(E)** Representative Alcian blue and TTC staining showing comparable ischemic regions between DMSO (Ctr) and 5SM-treated hearts at 7 days after MI. Scale bar, 1 mm. **(F)** Quantitative analysis of the data from panel **E**. n = 3 rats/group; Data are mean ± SEM.; ns, no significant; unpaired, two-tailed Student's *t*-test.

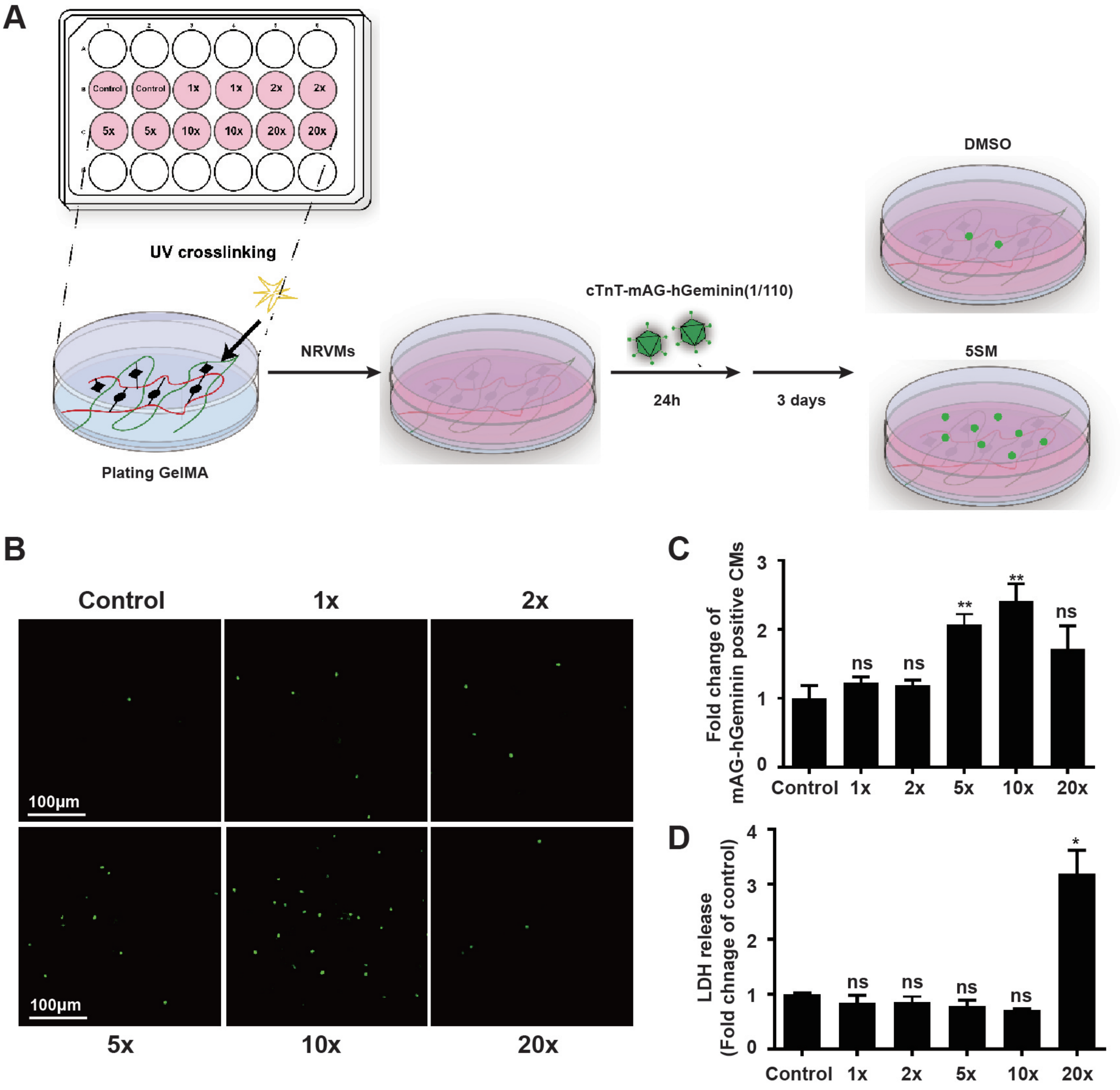

Supplementary Figure S2. Determination of the optimal dose of 5SM delivered by GelMA hydrogel. (A) Schematic diagram of Control and different doses of 5SM by GelMA hydrogel delivery in neonatal rat ventricular myocytes (NRVMs) for determining the optimal delivery dose *in vivo*. **(B and C)** The representative images **(B)** and statistics **(C)** of FUCCI positive NRVMs after treating with DMSO or different doses of 5SM for 3 days. n = 3,000 cells per replication, 3 replicates per group. Scale bars, 100 μm. Data are mean ± SEM.; ns, not significant; $^{**}p<0.01$; one-way ANOVA followed by Dunnett's multiple comparisons test. **(D)** The LDH levels of NRVMs were assessed after treating with DMSO or different doses of 5SM for 3 days. n = 3,000 cells per replication, 3 replicates per group. Data are mean ± SEM.; ns, not significant; $^{*}p < 0.05$; one-way ANOVA followed by Dunnett's multiple comparisons test. 5SM (1x) include norepinephrine hydrochloride (2

μmol/L), baricitinib (4 μmol/L), harmine (5 μmol/L), VO-OHpic trihydrate (2 μmol/L), and AZD3965 (2 μmol/L).

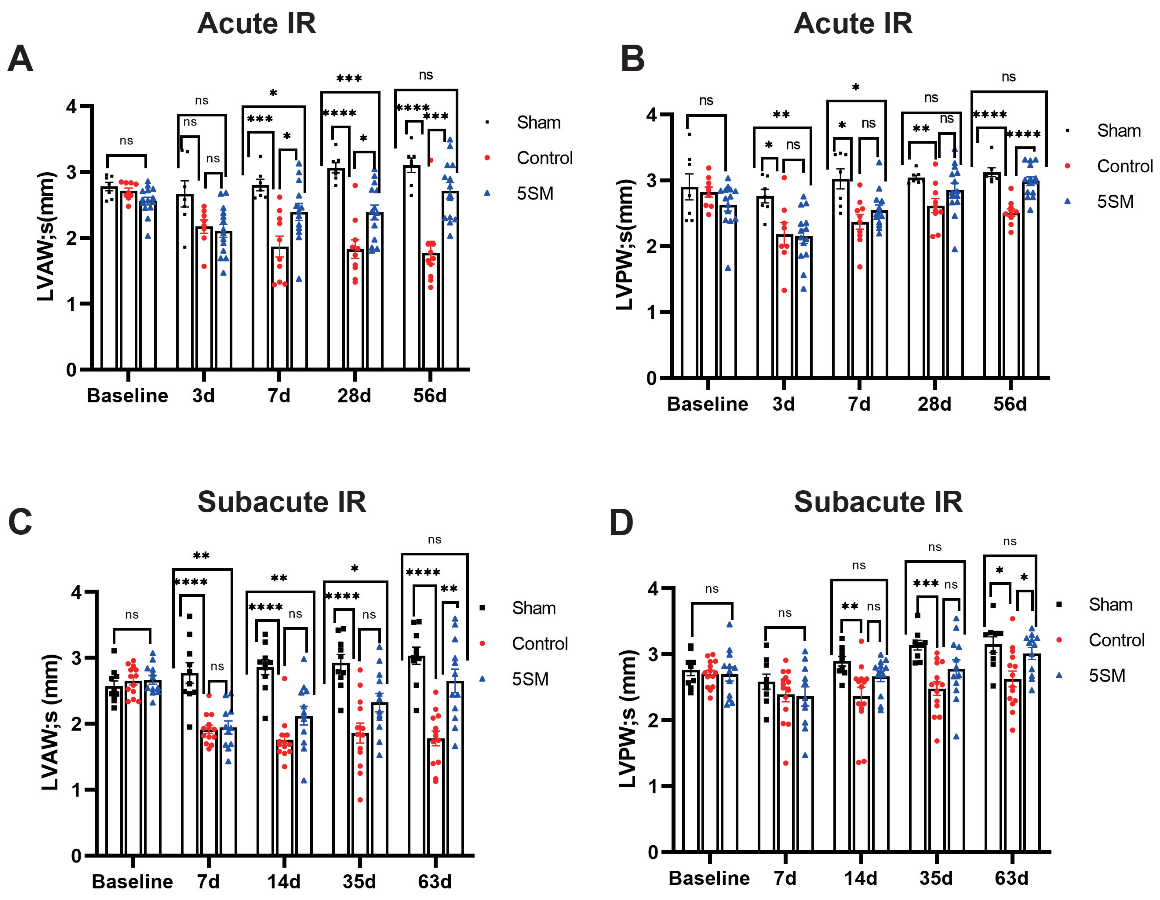

Supplementary Figure S3. 5SM improved cardiac function after acute and subacute IR injury. (A and B) Serial ECHO measurements of LVAW;s **(A)**, and LVPW;s **(B)** of Sham, Control (DMSO), and 5SM groups after acute IR. Sham, n = 7; Control, n = 9; 5SM, n = 13. Data are the mean ± SEM.; ns, not significant; *p <0.05; **p <0.01; ***p <0.001; ****p <0.0001; two-way ANOVA followed by Tukey's multiple comparisons test. **(C and D)** ECHO data showing that LVAW;s **(C)** and LVPW;s **(D)** increased after 5SM treatment in the subacute IR group. Sham, n = 9; Control, n = 14; 5SM, n = 12 rats. Data are the mean ± SEM.; ns, not significant; *p <0.05; **p <0.01; ***p < 0.001; ****p <0.0001; two-way ANOVA followed by Tukey's multiple comparisons test.

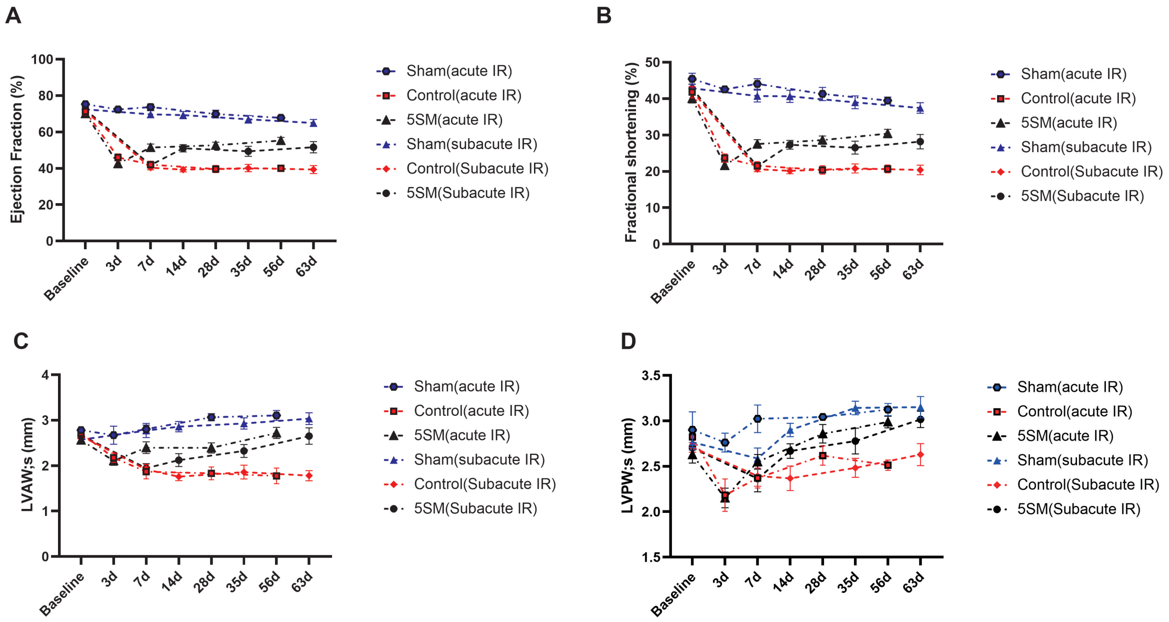

Supplementary Figure S4. Similar effects of 5SM on cardiac function took place after acute-IR and subacute-IR injury at different time points. (A and B) Serial ECHO measurements of ejection fraction **(A)** and fractional shortening **(B)** of sham, Control (DMSO), and 5SM groups after acute and subacute IR. Acute-IR: Sham, n = 7; Control, n = 9; 5SM, n = 13; Subacute-IR: Sham, n = 9; Control, n = 13; 5SM, n = 15 rats. **(C and D)** Serial ECHO measurements of LVAW;s **(C)**, and LVPW;s **(D)** of Sham, Control (DMSO), and 5SM groups after acute and subacute IR. Acute-IR: Sham, n = 7; Control, n = 9; 5SM, n = 13; Subacute-IR: sham, n = 9; Control, n = 13; 5SM, n = 15 rats.

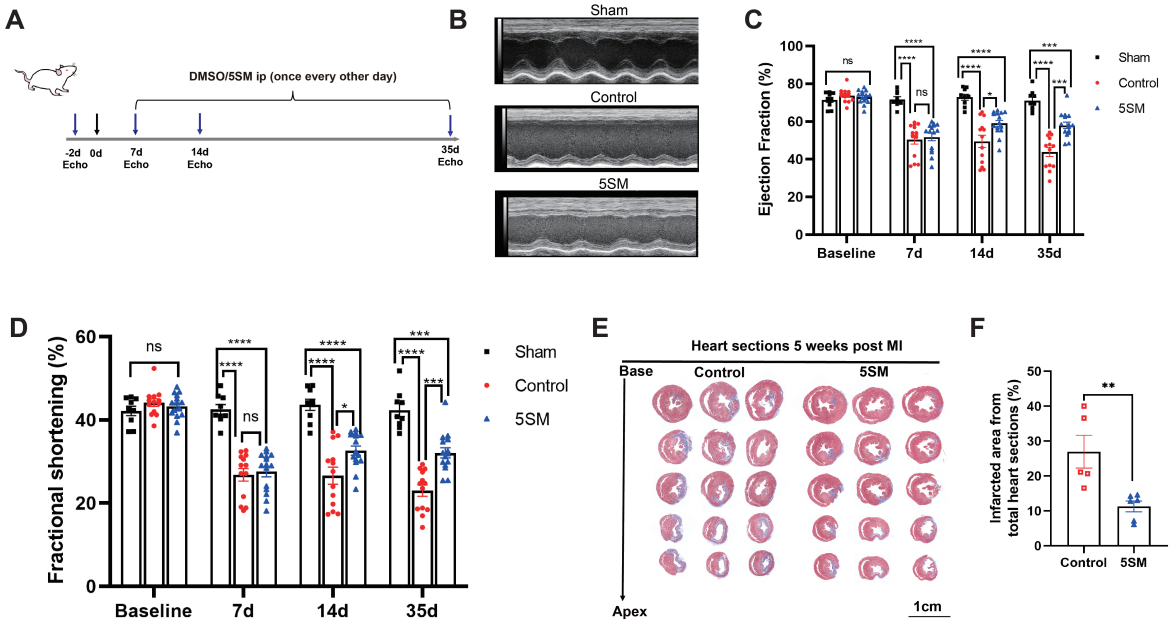

Supplementary Figure S5. 5SM improved cardiac function and decreased cardiac fibrosis after subacute MI injury. (A) Schematic showing DMSO or 5SM delivery, ECHO, and heart harvests in adult rats after subacute MI. **(B)** Representative images of M-mode ECHO from Sham, Control (DMSO), and 5SM-treated rats at 35 days post-MI. Heart rates were controlled to be similar in the three groups. **(C and D)** Serial ECHO measurements of ejection fraction **(C)** and fractional shortening **(D)** of Sham, Control (DMSO), and 5SM groups after subacute-MI. Sham, n = 9; Control, n = 13; 5SM, n = 15 rats. Data are the mean ± SEM.; ns, not significant; *p <0.05; ***p <0.001; ****p <0.0001; two-way ANOVA followed by Tukey's multiple comparisons test. **(E and F)** Masson's staining of heart sections from DMSO- and 5SM-treated adult rats at 35 days after subacute-MI **(E)** and quantitative analysis of cardiac fibrosis **(F)**. Control, n = 5; 5SM, n = 6 rats. Data are the mean ± SEM.; **p <0.01; unpaired, two-tailed Student's *t* test. Scale bar, 1 cm. Fibrotic scar was calculated relative to total section area.

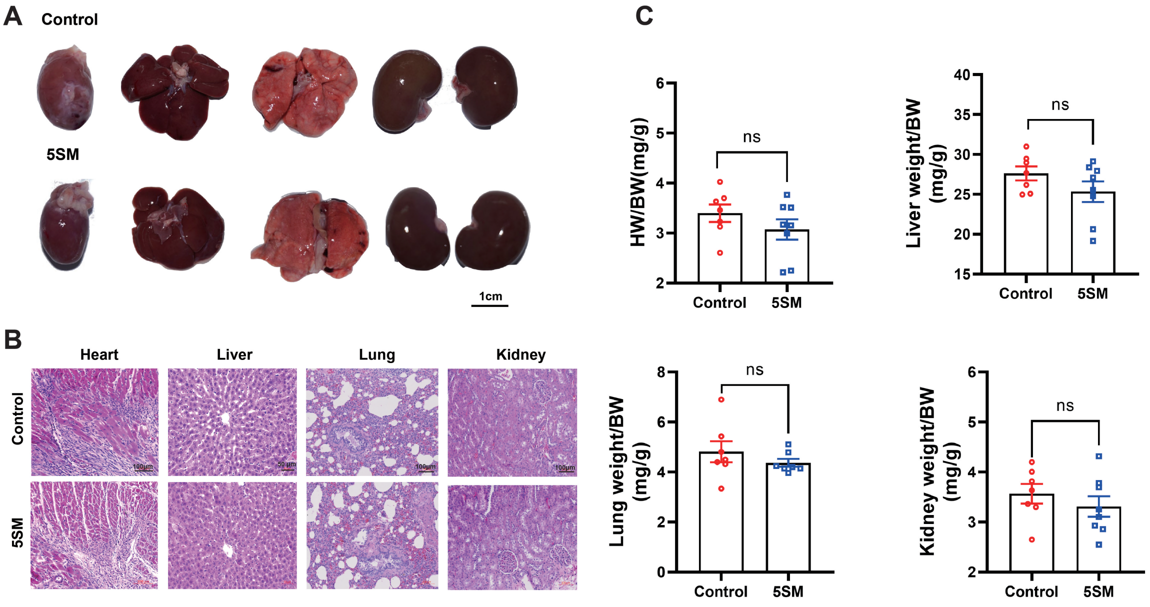

Supplementary Figure S6. The morphology of organs and organ weight to body weight showed no significant difference between Control and 5SM groups. (A) The morphology of heart, liver, lung and kidney in Control and 5SM groups. Scale bar, 1 cm. **(B)** HE staining images of heart, liver, lung and kidney in Control and 5SM groups. Scale bars of heart, lung and kidney, 100 μm; Scale bar of liver, 50 μm. **(C)** Statistics of heart weight (HW) to body weight (BW) ratio, liver weight to body weight ratio, lung weight to body weight ratio, and kidney weight to body weight ratio in Control and 5SM groups. n = 5 rats. Data are the mean ± SEM.; ns, not significant; unpaired, two-tailed Student's *t* test.
